# Supplementary material for: Lexical Frequency and Sentence Context Influence the Brain’s Response to Single Words
Source: Neurobiol Lang (Camb). 2022 Feb 10;3(1):149–79. doi: 10.1162/nol_a_00054 (PMC10158670; doi:10.1162/nol_a_00054)

Lexical frequency and sentence context influence the brain's response to single words
Supplementary material

Eleanor Huizeling, Sophie Arana, Peter Hagoort, Jan Mathijs Schoffelen

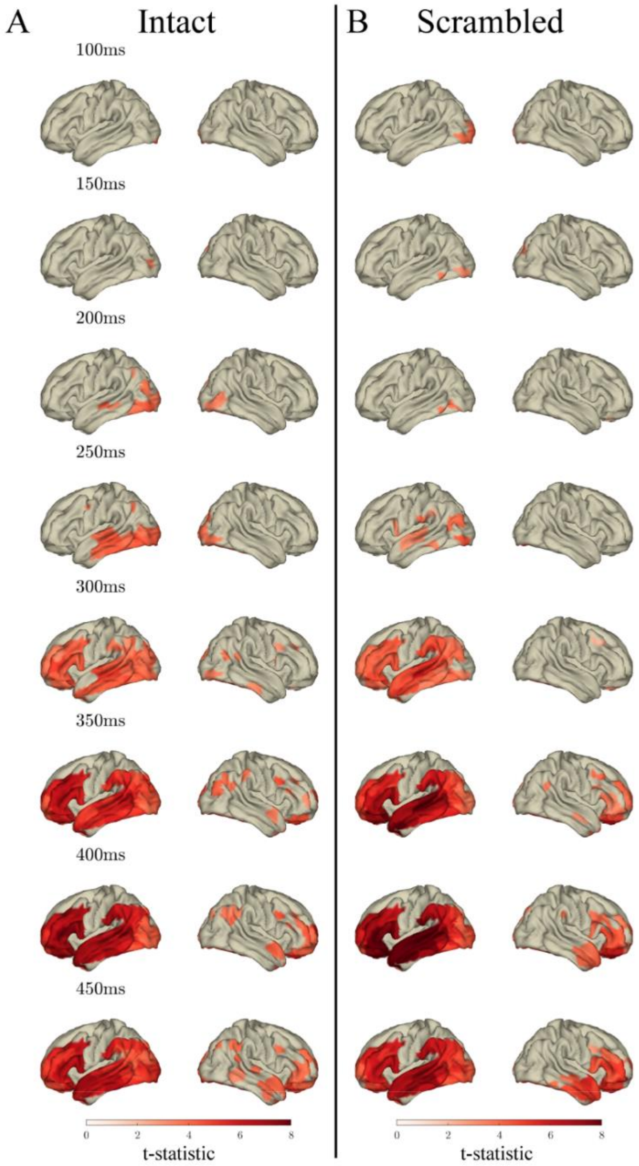

Figure SM1. Effects of lexical frequency in the response to content words: Surface plots of T-statistics (averaged over 50ms time windows centred at the indicated latencies, for visualisation) quantifying the difference in variance explained by lexical frequency (log10 transformed), beyond that explained by index, surprisal (log10 transformed), entropy, length, bigram letter frequency (log10 transformed) and trigram letter frequency (log10 transformed) in intact sentence compared to random permutation models (panel A; $p<.05$ one-sided, corrected) and scrambled sentence compared to random permutation models (panel B; $p<.05$ one-sided, corrected). Parcels for which no time point was significant during the 50ms time bin are masked.

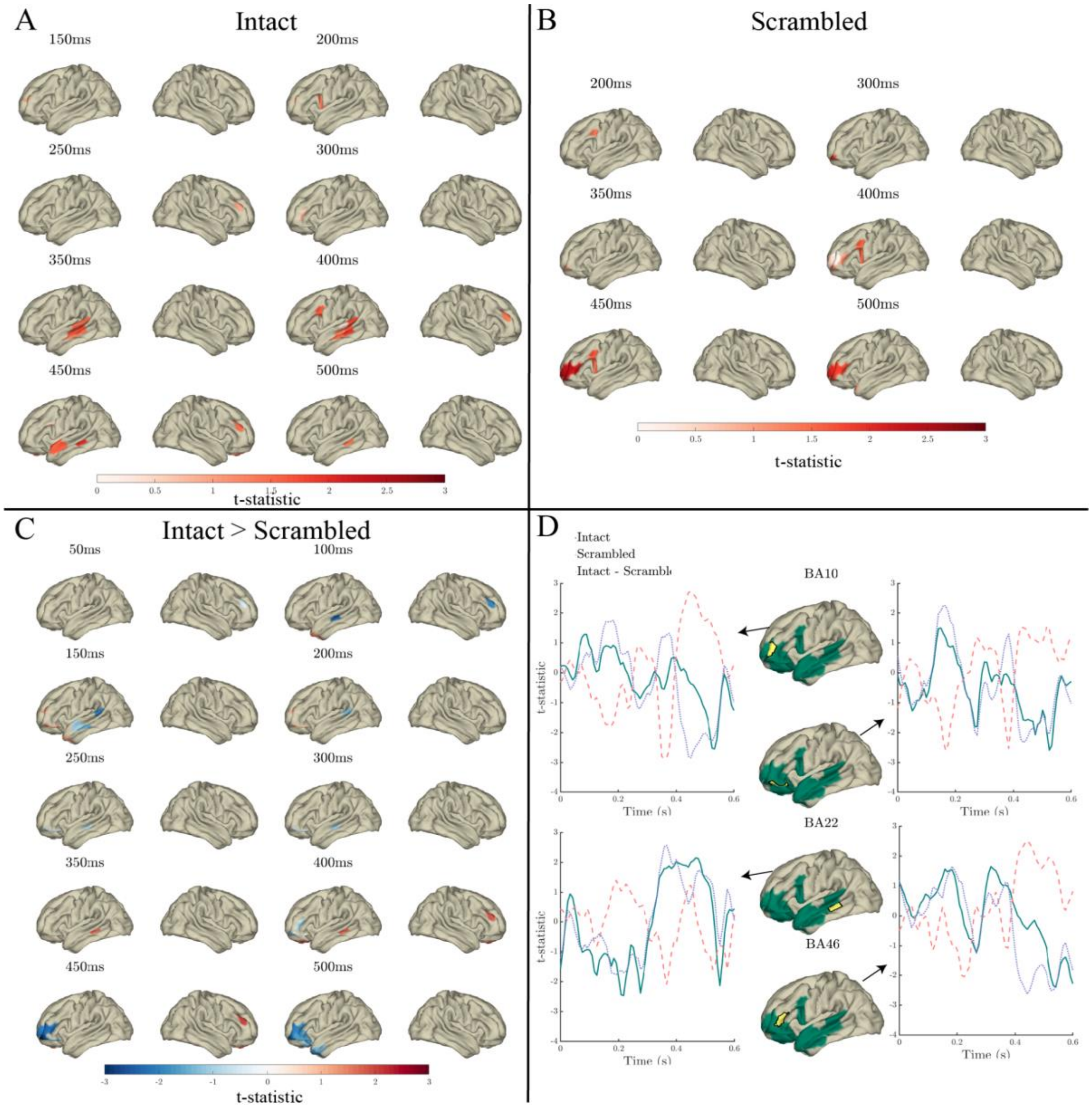

Figure SM2. Effects of the lexical frequency × index interaction in the response to content words: Surface plots of T-statistics (averaged over 50ms time windows centred at the indicated latencies, for visualisation) quantifying the difference in variance explained by lexical frequency × index interaction, beyond that explained by lexical frequency (log10 transformed), index, length, bigram letter frequency (log10 transformed) and trigram letter frequency (log10 transformed) in intact sentence compared to random permutation models (panel A; $p<.05$ one-sided, uncorrected), scrambled sentence compared to random permutation models (panel B; $p<.05$ one-sided, uncorrected), and intact compared to scrambled sentence models (panel C; $p<.05$ two-sided, corrected). Parcels for which no time point was significant during the 50ms time bin are masked. Panel D: Time courses of T-statistics for intact (solid green line) and scrambled (dashed red line) sentence models compared to random permutation models, and intact compared to scrambled sentence models (dotted purple line) for subparcels of BA10, BA11 BA22 and BA46 (highlighted in yellow on adjacent surface plots). ROIs entered into statistical analyses are illustrated as green shaded areas on surface plots.

Figure

Click here to access/download;Figure;Fig1.tif

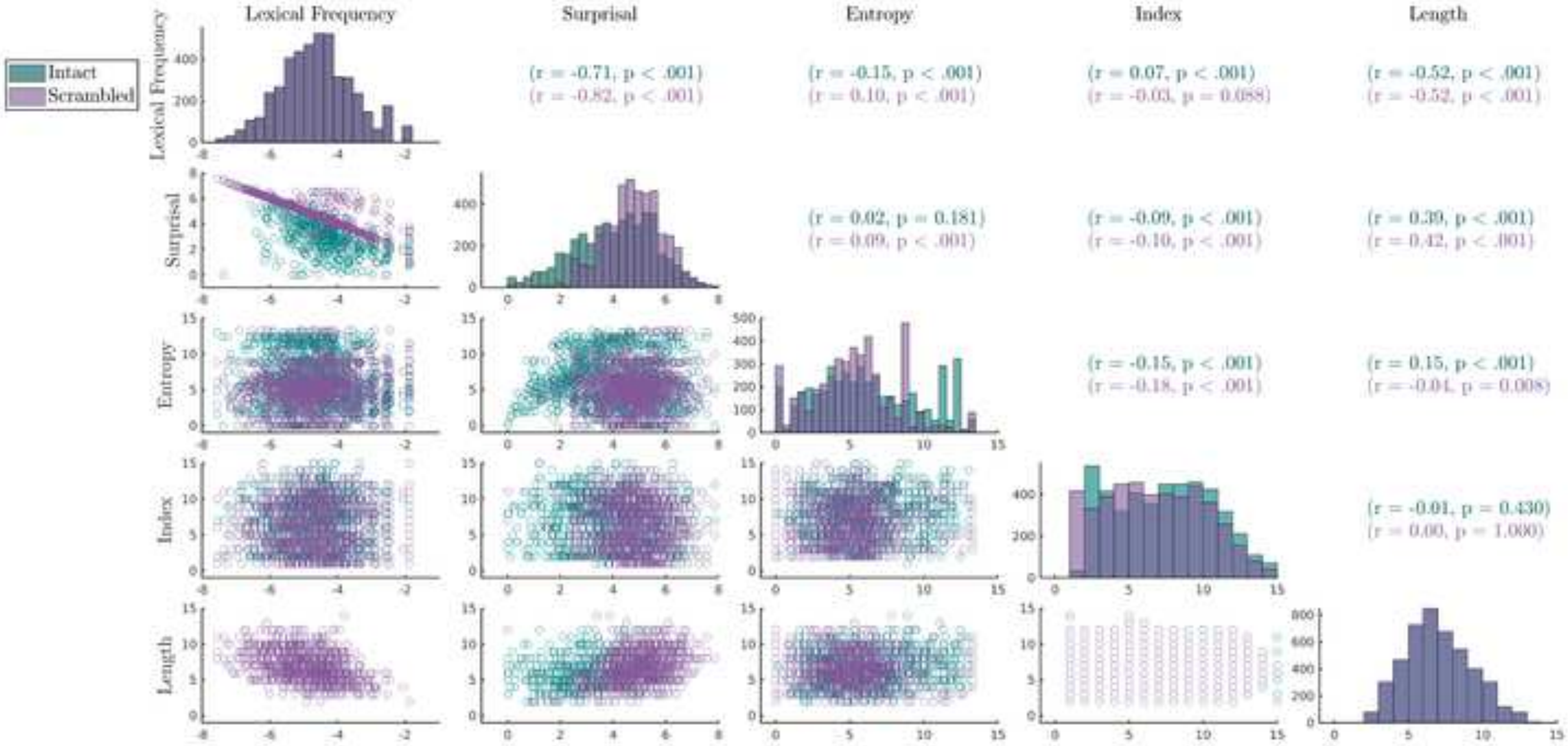

Figure

Click here to access/download;Figure;Fig2.tif

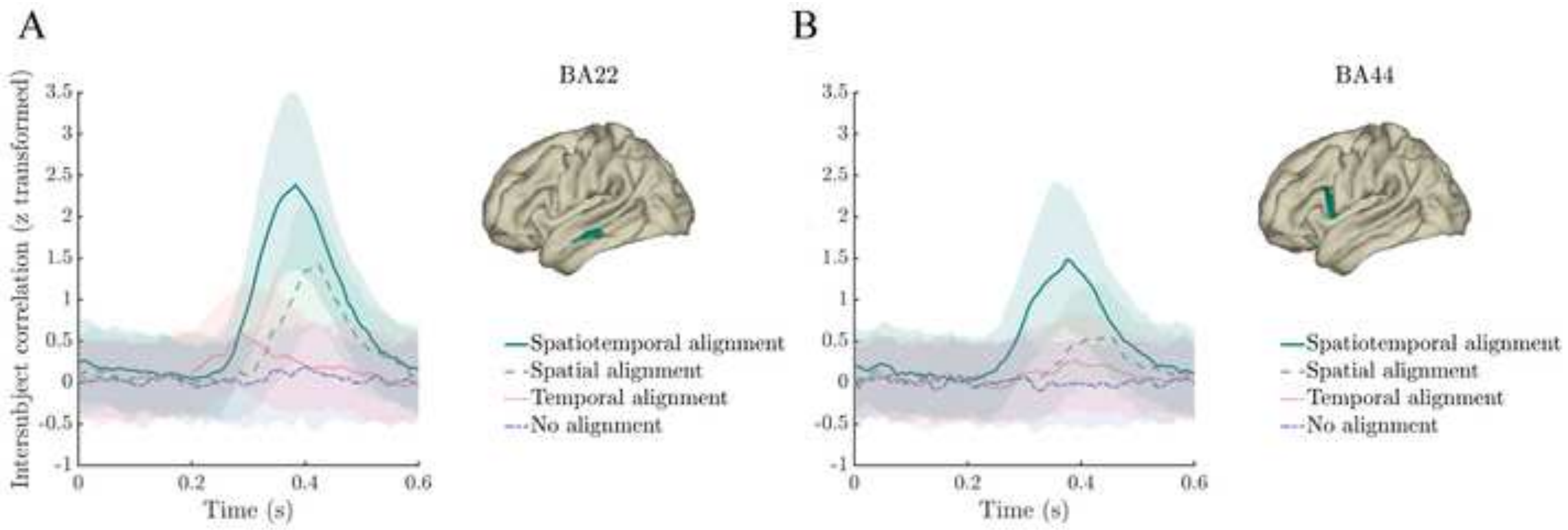

Figure

Click here to access/download;Figure;Fig3.tif

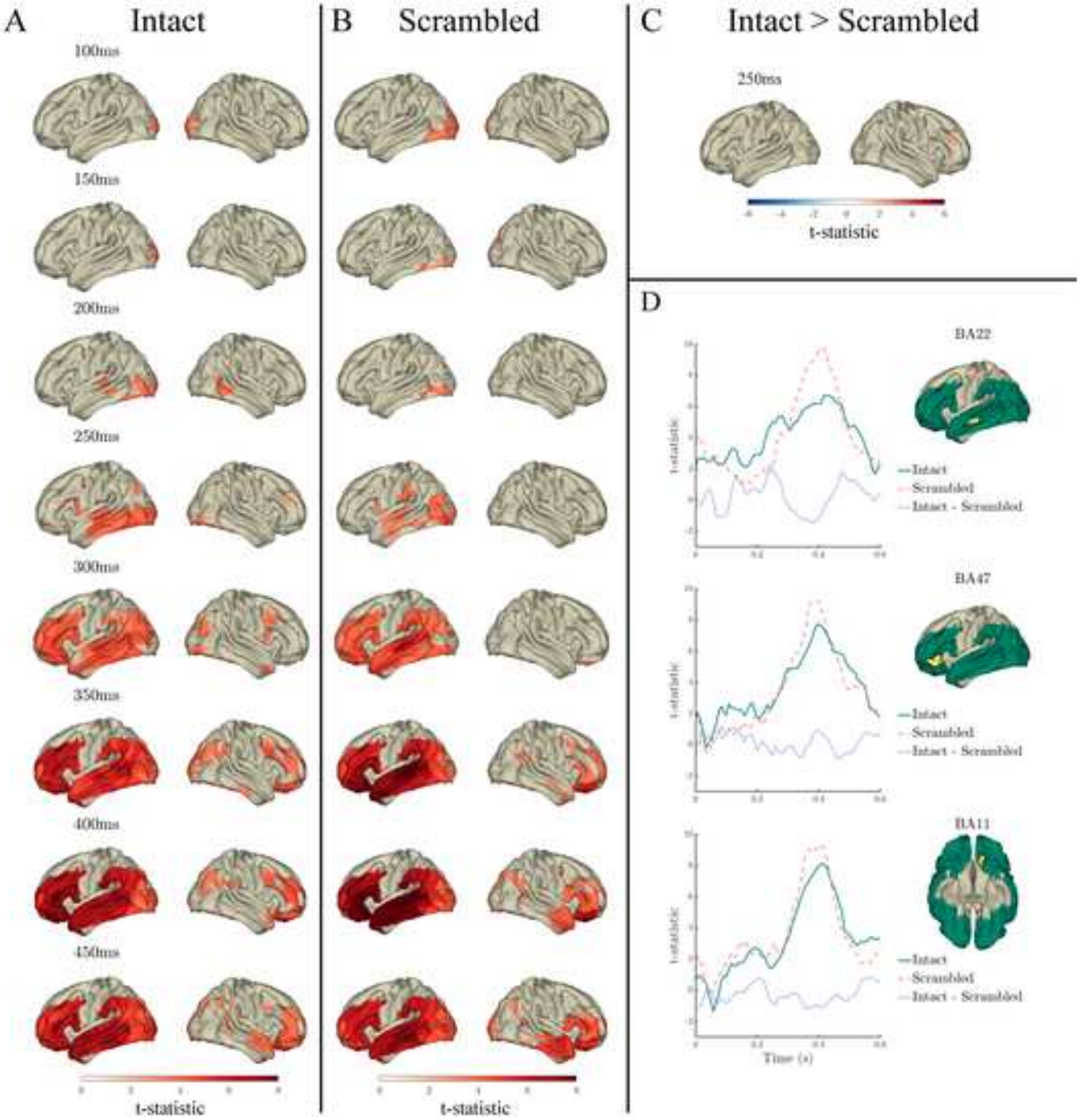

Figure

Click here to access/download;Figure;Fig4.tif

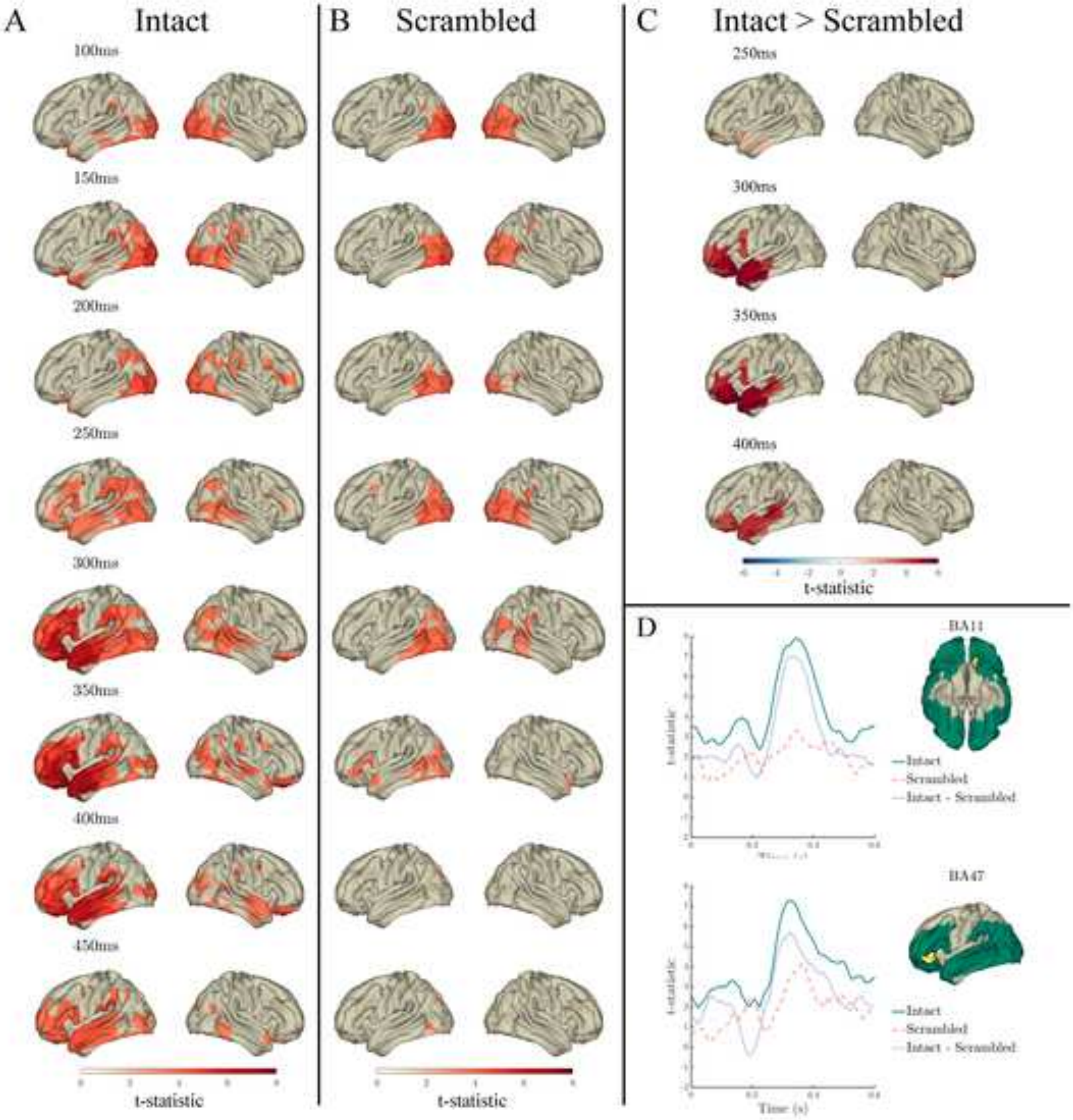

Figure Click here to access/download;Figure;Fig5.tif

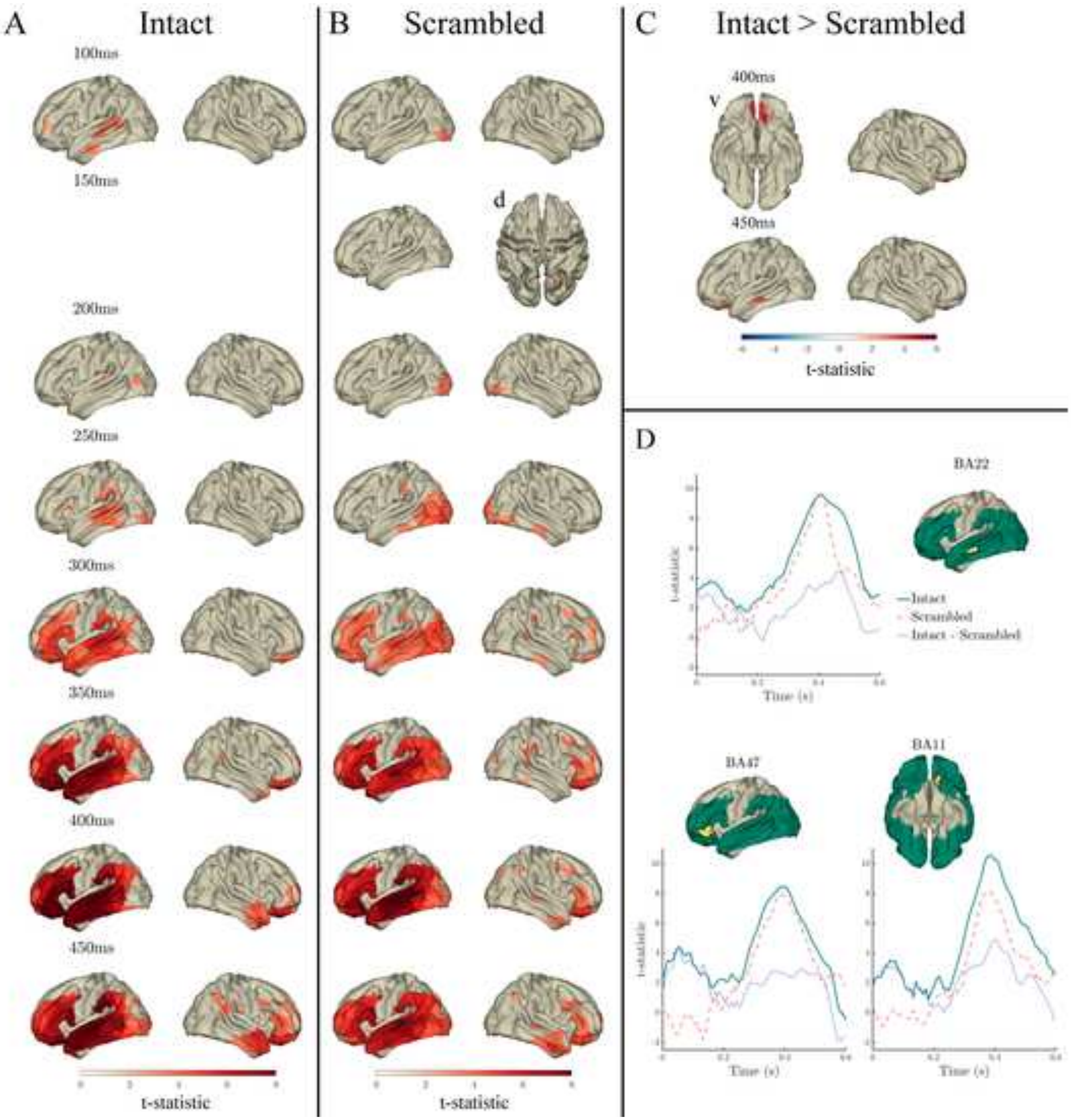

Figure
Click here to access/download;Figure;Fig6.tif

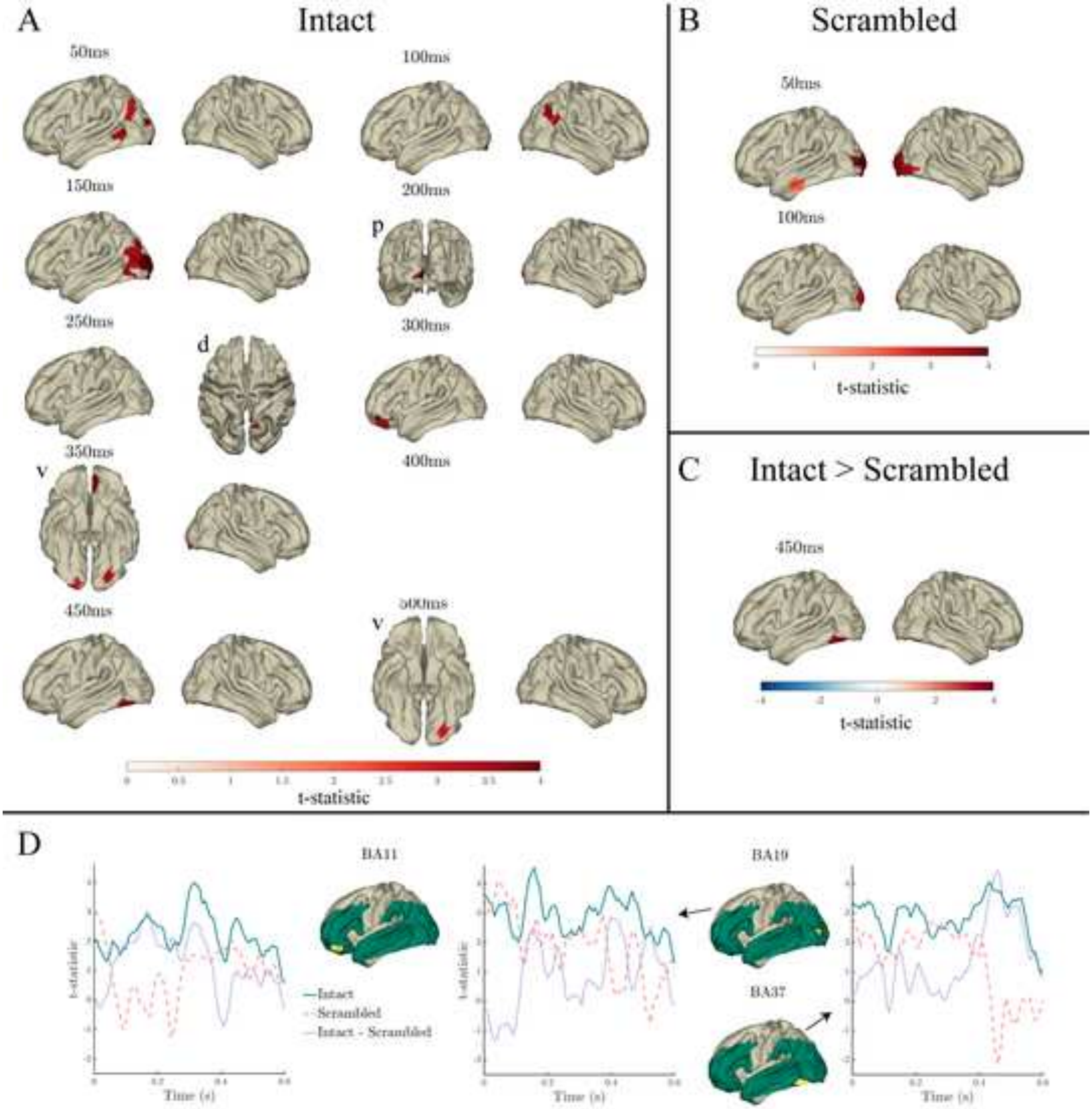

Figure Click here to access/download;Figure;Fig7.tif

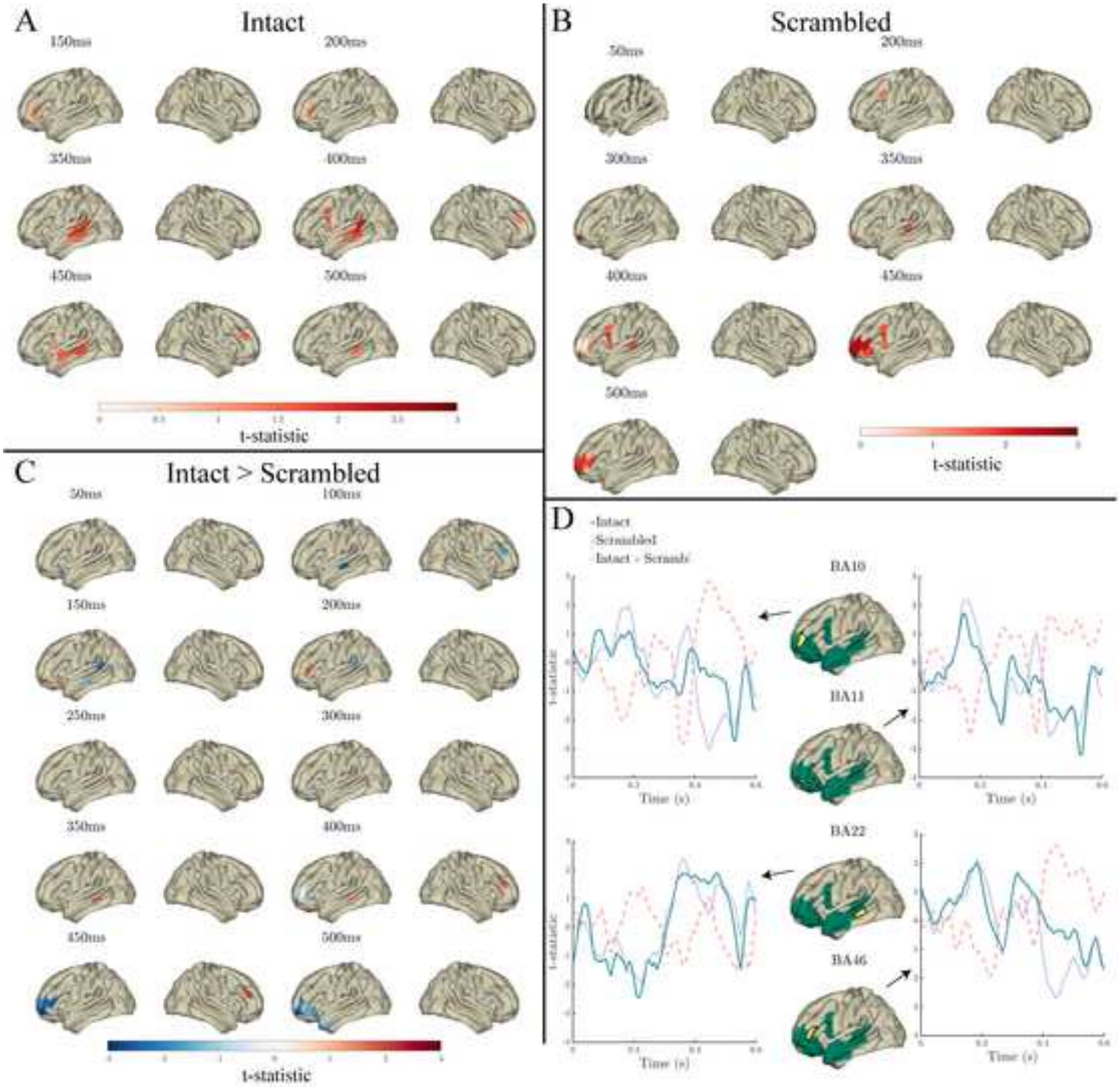

Figure Click here to access/download;Figure;Fig8.tif

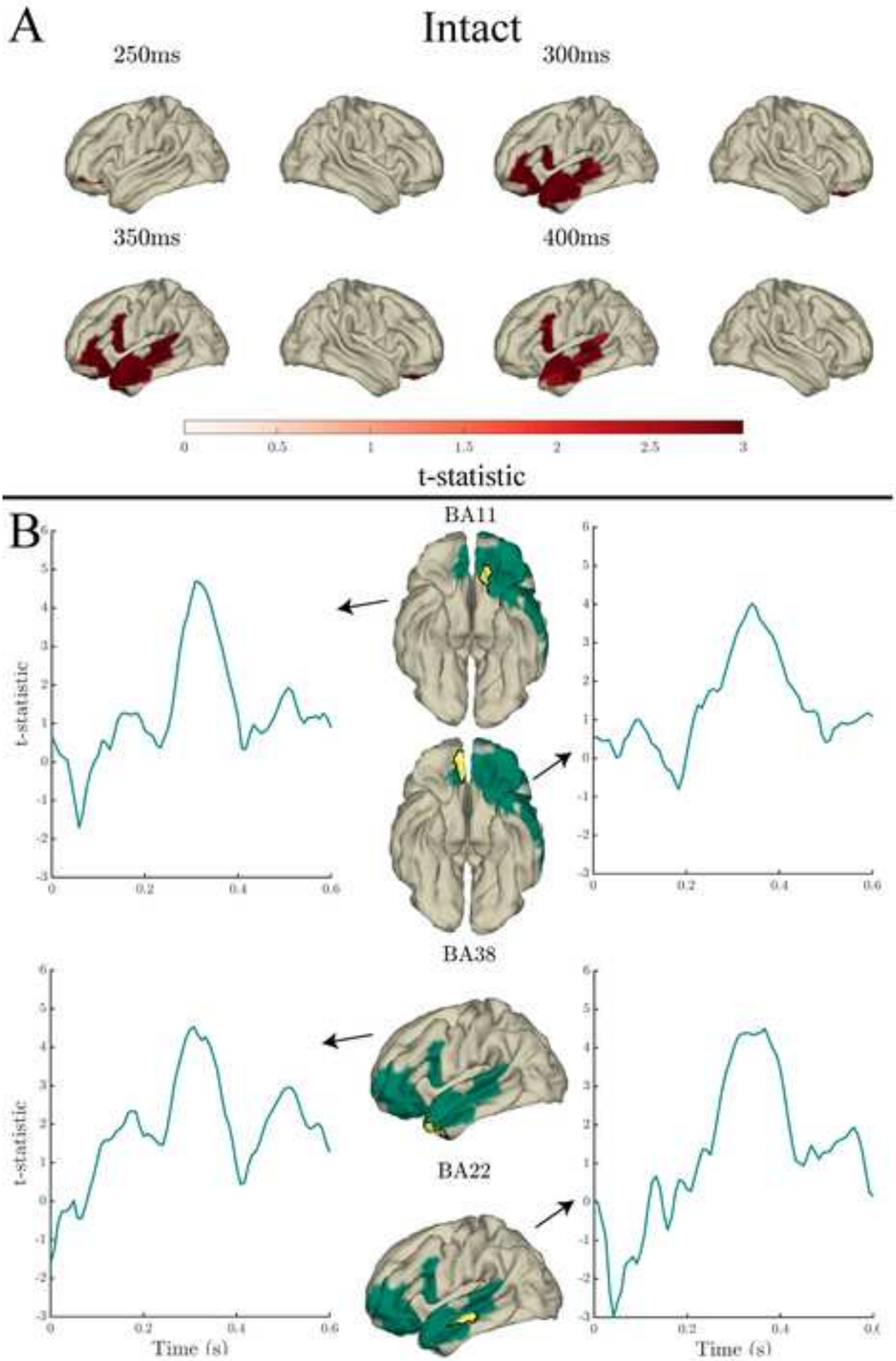

Figure

Click here to access/download;Figure;Fig9.tif

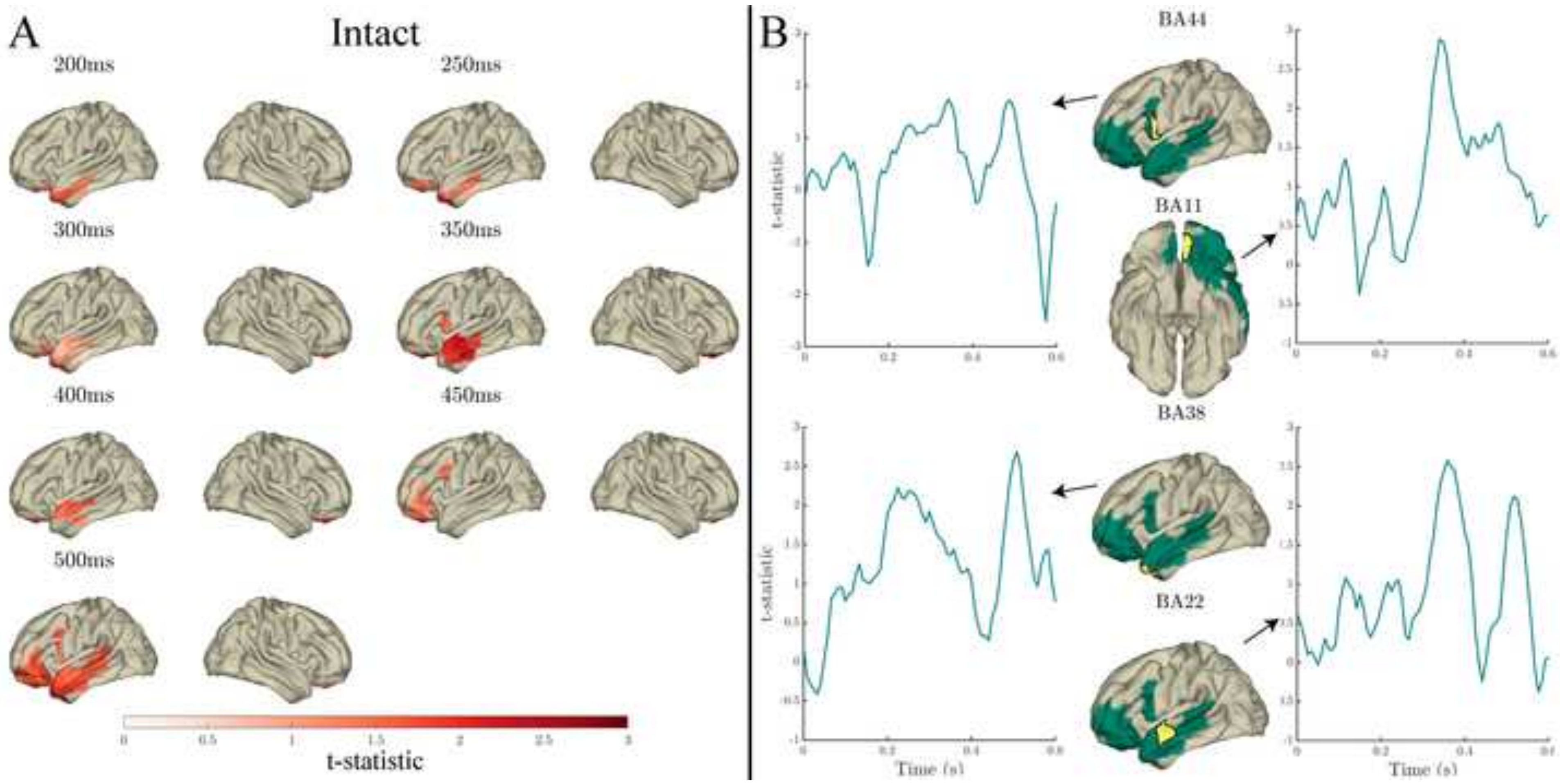

Supplement: Supplementary file 1 [file nol-3-1-149-s001.pdf]
